# Stakeholders’ views on online interventions to prevent common mental health disorders in adults implemented into existing healthcare systems in Europe

**DOI:** 10.1093/eurpub/ckab043

**Published:** 2021-07-07

**Authors:** Stefanie Kuso, Martina Nitsch, Michael Zeiler, Monika Simek, Tanja Adamcik, Michelle Dey, Thomas Berger, Tobias Krieger, Kiona K Weisel, Anna-Carlotta Zarski, David D Ebert, Michael P Schaub, Christian T Moser, Christina Botella, Rosa Baños, Rocio Herrero, Ernestina Etchemendy, Barbara Nacke, Ina Beintner, Bianka Vollert, Juliane Schmidt-Hantke, Kristian Hütter, Corinna Jacobi, Karin Waldherr

**Affiliations:** 1 Ferdinand Porsche FernFH—Distance Learning University of Applied Sciences, Wiener Neustadt 2700, Austria; 2 Department for Child and Adolescent Psychiatry, Medical University of Vienna, Vienna 1090, Austria; 3 Swiss Research Institute for Public Health and Addiction ISGF, Associated to the University of Zurich, Zurich, Switzerland; 4 Department of Clinical Psychology and Psychotherapy, University of Bern, Bern, Switzerland; 5 Department of Clinical Psychology and Psychotherapy, Friedrich-Alexander University Erlangen-Nürnberg, Erlangen, Germany; 6 Jaume I University, Castellón, Spain; 7 CIBER Pathophysiology of Obesity and Nutrition (CB06/03), Carlos III Institute of Health, Madrid, Spain; 8 Department of Personality, Evaluation and Psychological Treatments, University of Valencia, Valencia, Spain; 9 Department of Clinical Psychology and Psychotherapy, TU Dresden, Dresden, Germany

## Abstract

**Background:**

Online preventive interventions can help to reduce the incidence of mental disorders. Whereas knowledge on stakeholders’ attitudes and factors relevant for successfully integrating online treatment into existing healthcare systems is available, knowledge is scarce for online prevention.

**Methods:**

Stakeholders from Germany, Switzerland, Austria and Spain were surveyed. Potential facilitators/delivery staff (e.g. psychologists, psychotherapists) completed an online questionnaire (*n* = 183), policy makers (i.e. from the governing sector or health insurance providers) participated in semi-structured interviews (*n* = 16) and target groups/potential users of mental illness prevention (*n* = 49) participated in ten focus groups. Thematic analysis was used to identify their experiences with and attitudes and needs regarding online programmes to prevent mental disorders. Additionally, it was examined which groups they consider underserved and which factors they consider as fostering and hindering for reach, adoption, implementation and maintenance (cf. RE-AIM model) when integrating online prevention into existing healthcare systems.

**Results:**

Main advantages of online mental illness prevention are perceived in low structural and psychological barriers. Lack of personal contact, security, privacy and trust concerns were discussed as disadvantages. Relevant needs are high usability and target group appropriateness, evidence for effectiveness and the use of motivational tools.

**Conclusions:**

Positive attitudes among stakeholders are the key for successful integration of online mental illness prevention into existing healthcare systems. Potential facilitators/delivery staff must receive training and support to implement these programmes; the programmes must be attractive and continuously evaluated, updated and promoted to ensure ongoing reach; and existing infrastructure and contextual factors must be considered.

## Introduction

European healthcare systems are facing enormous challenges at various levels, whereby mental disorders are regarded as one of the most pressing issues with 13% of the global burden of disease attributable to them.^1^ Mental disorders are not only steadily increasing but also linked with preventable, non-communicable diseases.^2^ Accordingly, public spending on mental healthcare is on the rise, with e.g. treatment of mood disorders (bipolar disorder and major depression) being estimated to be the most cost-intensive category of mental disorders in Europe.^3^

A possible solution to the problem is the strengthening of prevention initiatives focussing on common mental health disorders, e.g. depression, anxiety disorders, eating disorders or substance use disorders. Prevention includes universal prevention targeting whole populations independent of specific risk factors, as well as selected (for persons with an elevated risk for mental health problems) and indicated prevention (for persons with subclinical mental health disorders).^4^ The Internet has been proven as suitable delivery medium for mental health interventions, which is regarded to have some advantages related to accessibility, flexibility, anonymity and cost-effectiveness in comparison to face-to-face interventions.^4^ Digital solutions for mental illness prevention may include unguided programmes mainly providing information or guided/moderated programmes (e.g. with psychoeducational and interactive elements and synchronous or asynchronous contact)^5^ which are theory-based (e.g. cognitive behavioural approach) and which are usually delivered via interactive websites or mobile applications. Although evidence for the prevention^4^^,^^6^ and treatment^7^ of mental health problems by means of digital solutions is accumulating, their implementation and actual use in routine care remains limited.^8–11^ Several explanatory approaches exist, however, most of them point to the fact that barriers for implementation have a significant impact on the uptake of online mental health interventions. From a users’ perspective, hindering factors regarding the implementation of digital healthcare solutions generally concern the workability of systems, e.g. ease of use, confidence, security and accountability. Moreover, a lack of understanding of potential aims and benefits of eHealth services and missing efforts of engaging potential users as well as the absence of knowledge regarding monitoring and evaluation of eHealth systems need to be considered.^12^

However, most of the existing studies only refer to treatment and not the prevention of mental health problems. More specifically in eMental health, barriers to the use of online programmes from users’ perspective must be considered of which negative attitudes towards such programmes, doubts about their effectiveness, a preference for face-to-face interventions and the lack of synchronous communication with health professionals seem to be most important.^8^^,^^10^^,^^13^^,^^14^ Furthermore, professionals’ perspectives regarding organizational structures and procedures, resources and leadership also have to be taken into account. In a recent interview study on the implementation of eMental healthcare with key stakeholders (academic, government, health organizations, and industry representatives), statements related to funding, credibility, knowledge gaps and patient empowerment were most prominent.^15^ Recently, a comprehensive survey on Internet-based cognitive behavioural therapy for depression was carried out in eight European countries including various stakeholder groups (care and technology providers, researchers, funders, government bodies and organizations representing patients/users) as part of the E-COMPARED project.^16^ Overall, stakeholders mentioned cost-efficiency as the main advantage and low feasibility of delivery as the main barrier to implementation of Internet-based solutions into existing regular healthcare systems.^16^ However, determinants for the successful implementation of digital interventions aiming to prevent mental health problems might differ from those relevant for treatment and eHealth in general. Thus, the reported implementation facilitators and barriers cannot be generalized to preventive interventions.

Hence, the aim of this article is to provide a comprehensive overview on stakeholders’ experiences with, attitudes and needs regarding online interventions to prevent mental disorders as well as their view on context factors influencing the implementation and dissemination of online mental illness prevention in existing healthcare systems in Europe.

## Methods

This study is part of the stakeholder survey conducted in the course of the European project ‘ICare’.^17^ The aim of ICare (https://www.icare-online.eu, 25 March 2021, date last accessed) was to implement a variety of online interventions targeting common mental health problems for different target groups (TGs) in three settings (healthcare, school, university) across six European countries.^18^ This study focuses on the healthcare setting. A detailed description of the study design of the ICare stakeholder survey and results of the other settings are published in separate papers in this supplement.^17^^,^^19–22^

### Design and instruments

We sought to include the perspectives of diverse key stakeholders to investigate factors relevant for implementation, dissemination and exploitation of ICare interventions. In accordance with the overall design of the ICare stakeholder survey,^17^ three stakeholder groups in the healthcare setting were approached using a concurrent mixed-methods design: (i) An anonymous online questionnaire was sent to potential facilitators/delivery staff of online mental illness prevention. Facilitators/delivery staff are relevant occupational groups for fostering the delivery of online mental illness prevention programmes, e.g. psychologists, psychotherapists, psychiatrists and general practitioners. (ii) Semi-structured interviews were held with policy makers (PMs). PMs contribute to decision-making processes on the system level and are therefore relevant for a sustainable implementation of prevention programmes in the healthcare setting. (iii) Focus groups were conducted with target groups/potential users (TGs) according to the characteristics of the ICare interventions implemented in European healthcare systems (‘EveryBody’^23^ and ‘ICare Prevent’^24^). Since the respective ICare interventions tackle different mental health disorders and different stages of prevention, TGs /potential users ranged from people with no symptoms at all to people with subclinical symptoms of depression and anxiety. Thus, individuals with characteristics which make them potential users of these two ICare online prevention programmes were approached. For ‘EveryBody’ participants in the healthcare system in Germany (GER), and for ‘ICare Prevent’ in GER, Switzerland (CH), and Spain (ES) were recruited. The recruitment involved pre-assessments according to the inclusion and exclusion criteria of the respective ICare interventions, i.e. women above 18 without symptoms of an eating disorder for ‘Everybody’^23^ and participants with subclinical symptoms of anxiety and depression for ‘ICare Prevent’.^24^ Since Austria (AT) was not a partner in the trials, but rather a country in which additional recruitment took place, participants for the stakeholder survey in AT were only recruited on a non-intervention-specific basis by including adults above 18 years without any screening.

The items of the online questionnaire as well as the topic guides used for the focus groups and interviews (see the Supplementary material) were strongly oriented towards the research dimensions deduced from the reach, effectiveness, adoption, implementation, maintenance (RE-AIM) framework.^25^ The following main themes were addressed in all stakeholder groups: stakeholders’ experiences with online interventions to prevent mental health problems; attitudes towards online prevention (especially perceived advantages and disadvantages); needs (including required programme characteristics, topics, and aims); underserved groups (which might benefit most); as well as factors and context parameters that could foster or hinder reach, adoption, implementation and maintenance of online programmes to prevent mental disorders in the healthcare setting. Stakeholders received definitions of ‘prevention’, ‘Internet-based prevention programs’, ‘reach’, ‘adoption’, ‘implementation’ and ‘maintenance’ to foster a common understanding of these terms.

Ethical approval for this study was obtained from the relevant local ethics committees in all participating countries. All stakeholders gave their informed consent (including consent for audio recordings of focus groups and interviews).

### Recruitment and procedures

The recruitment of the survey participants took place in Austria, Germany, Spain and Switzerland and followed a criterion based sampling strategy described in more detail in Nitsch *et al*.^17^ Considering the concept of data saturation and standards for the field,^17^ we planned to conduct 4 interviews with PMs per country (16 interviews across all countries) and 10 focus groups with TGs/potential users of the respective ICare interventions across all countries. For the online questionnaire we planned to recruit a sample size of 20–30 potential facilitators/delivery staff per country (in total 80–120 respondents). A detailed recruitment plan and rationale for the planned sample size is published in the design paper of the ICare stakeholder survey in this Supplement.^17^

Following a criterion-based sampling strategy, consortium partners were asked to identify and approach PMs in their countries, taking different positions in the hierarchy of the healthcare system into account (i.e. representatives of Health Ministries, health insurance providers, care providers and research institutions as consultants of PMs). As the partners know the situation in their countries best, no further standards were set, but researchers were asked to collect background information on different interviewee characteristics (current position in the healthcare system, number of years of experience in the healthcare setting) and to judge the level of influence/power regarding adoption, implementation processes and maintenance of online prevention in mental health. Potential interview partners were invited via e-mail or telephone requests to take part in semi-structured interviews, which were conducted via telephone (nine interviews) or face-to-face (seven interviews) and audiotaped.

Potential facilitators/delivery staff were identified via official websites and mailing lists and were approached via e-mail and invited to complete the online questionnaire (e.g. in Austria an e-mail was sent to a random sample of members from the list of all licenced clinical psychologists and psychotherapists provided at the website of the Austrian Health Ministry; in Switzerland associations of social workers and psychotherapists were asked to send the invitation to their members). In the other countries the invitation was distributed via e-mails to diverse institutions concerned with mental health, via social media (e.g. Facebook) and diverse newsletters. Since participants were recruited via diverse channels in the different countries, including social media, the number of potentially reachable facilitators cannot be determined across all countries.

For the focus groups, participants were recruited by the research teams of the trials mainly via counselling centres, social media, notice boards (e.g. job posting sites) and e-mail distribution lists (e.g. lists of students of several universities and alumni associations). Pre-assessments were conducted via online surveys. Since we were not able to include a large enough number of participants fulfilling inclusion criteria of ‘ICare Prevent’ in Spain and Switzerland, the inclusion criteria had to be broadened, to allow participants with lower scores on the pre-assessments to participate. The focus groups were held at the universities’ facilities and were audiotaped. In Austria, focus group participants received gift cards of 25 Euros, in Germany 20 Euros, in Spain 10 Euros and in Switzerland 100 CHF as incentives for participation. Participating PMs and potential facilitators/delivery staff did not receive any compensation. Informed consent for audiotaping the interviews and focus groups was provided by the participants.

### Data analyses

The focus groups and interviews were transcribed verbatim in German and Spanish. Spanish transcripts and Spanish answers to open-ended questions of the online questionnaire were translated into German or English by members of the research team. The data were coded and categorized by Austrian researchers using NVivo 11 Pro software^26^ and a thematic analysis approach following Froschauer and Lueger^27^ was applied. A team of researchers (S.K. and M.S.) in Austria coded the transcripts and identified common themes and their characteristics (categories). The themes and categories were discussed and interpreted by the Austrian research team, until consent was reached. Deductive main categories were predetermined based on the research questions. The structure of the results part of this paper reflects these main categories. The subthemes were generated inductively. The open-ended questions of the online questionnaire were coded separately because they mostly consisted of catchwords or short phrases. However, the identified categories matched the categories in the coding system that emerged from the analysis of the transcripts. Since there were no major differences or contradictions in the emerging themes between stakeholder groups and countries, the overall qualitative results from the focus groups, the interviews and the open-ended questions of the online questionnaire are presented together in the Results section. Typical quotes from PMs, potential facilitators/delivery staff (F) and TGs/potential users are added for further illustration.

Descriptive statistics were used to describe answers to the closed questions about experiences, attitudes and needs of potential facilitators/delivery staff who completed the online questionnaire. Experiences with Internet-based prevention programmes in mental health as well as relevance of topics and characteristics were rated on a 10-point scale (0 = no experience whatsoever/not at all relevant, 10 = a lot of experience/very relevant) by the facilitators/delivery staff only. Furthermore, potential facilitators/delivery staff rated to what extent they would be in favour of integrating online prevention in the healthcare setting in their country and if they would actively support its integration (0 = not at all, 10 = absolutely) and how they would weigh advantages and disadvantages of online prevention programmes compared with face-to-face contacts (−5= many more disadvantages, +5= many more advantages, 0= neutral). Chi-square tests (for categorical variables) and Kruskal–Wallis tests (for continuous variables with skewed distributions) were used to explore differences between potential facilitators/delivery staff from the four participating countries regarding their experiences with and attitudes towards Internet-based prevention programmes in the field of mental health. The quantitative results from the online questionnaire filled out by the potential facilitators/delivery staff are presented in Supplementary table 2 and figures.

## Results

Across all four countries, we obtained 183 online questionnaires from potential facilitators/delivery staff (F), conducted 16 interviews with PMs and 10 focus groups involving a total of 49 participants representing the TGs/potential users of the individual programs (TG). The semi-structured interviews lasted from 32 to 63 min (mean: 43, SD = 9) and the focus groups between 31 and 105 min (mean: 65, SD = 27). The main characteristics of the sample are presented in table 1.

**Table 1:** Sample characteristics of included stakeholders of the healthcare setting per method

Focus groups (total *n *= 10)	
Number of focus groups per country	*n* (%)
Austria	2 (16.7)
Switzerland	2 (16.7)
Germany	4 (33.3)
Spain	2 (16.7)
Participants per gender	N (%)
Total	49 (100)
Females	38 (77.6)
Males	11 (22.4)
Semi-structured interviews (Total *n* = 16)	
Number of interviews per country	*n* (%)
Austria	4 (25.0)
Switzerland	4 (25.0)
Germany	4 (25.0)
Spain	4 (25.0)
Type of interview	*n* (%)
In-person	7 (43.8)
Telephone	9 (56.2)
Participants per gender	*n* (%)
Females	6 (37.5)
Males	10 (62.5)
Stakeholder group^a^	*n* (%)
Governing sector	6 (35.3)
Insurance	6 (35.3)
Care provider	4 (23.5)
Research	1 (5.9)
Years of experience in their function (years)	*n* (%)
0–5	3 (18.8)
6–10	5 (31.3)
11–20years	3 (18.8)
>20	4 (25)
Unknown	1 (6.3)
Online questionnaire (total *n* = 183)	
Number of individuals per country	*n* (%)
Austria	81 (44.3)
Switzerland	29 (15.8)
Germany	50 (27.3)
Spain	23 (12.6)
Function	*n* (%)
General practitioner	10 (5.5)
Paediatrician	1 (0.5)
Psychologist	77 (42.1)
Psychotherapist	66 (36.1)
Social Worker	10 (5.5)
Psychiatrist	2 (1.1)
Nurse	2 (1.1)
Other medical specialist	3 (1.6)
Other	12 (6.6)
Years of experiences in their function	Mean (SD)/median (range)
Years	11.71 (9.3)/9.0 (0–40)

a As one individual has overlapping functions, the number don’t sum up to 16.

The following results of the thematic analysis include results from the focus groups (with the TGs/potential users) and interviews (with PMs) and the open-ended questions of the online questionnaires (filled out by the potential facilitators/delivery staff). Apart from the experiences with online mental illness prevention programmes, there were no differences across countries or stakeholder groups regarding the emerging themes. Therefore, the results are presented along the research questions, themes and subthemes, rather than per country or stakeholder group. The quantitative results refer exclusively to the closed questions of the online questionnaire for potential facilitators/delivery staff.

### Experiences

The interviewed PMs had different degrees of experiences with online mental illness prevention. Some had no experiences at all, others described programmes in whose development and/or implementation they were involved, some have heard about such programmes.

In the focus group discussions with the different TGs/potential users of the ICare interventions, mobile applications (apps) and Internet forums were perceived and discussed as online prevention interventions, with a wide variety of topics from meditation and healthy lifestyle to all kinds of mental disorders. As offline prevention they had experiences with, they named campaigns, relaxation techniques, workplace health promotion and others.

Of the potential facilitators/delivery staff (F) who participated in the online survey, 78.5% had already read or heard about, 44.8% had already looked at and 20.9% have already implemented online mental illness prevention programmes in the healthcare setting. Highest experience levels were reported in Switzerland and Spain, whereas lower experiences were reported in Austria and Germany (cf. Supplementary table 2). On average, overall experiences were rated low on the 10-point rating scale (mean: 2.36, SD = 2.76, median = 1). However, they also differed significantly between the countries with lowest ratings in Austria and highest in Switzerland (Kruskal–Wallis *H* = 20.84, *P* < 0.001).

### Attitudes

To examine all stakeholders’ attitudes against online mental illness prevention, we asked them which advantages and disadvantages of online prevention programmes in mental health they would anticipate. Across all stakeholder groups (F, PM and TG) we identified four themes regarding anticipated advantages and two themes regarding disadvantages.

#### Advantages

##### Low structural barriers

Online prevention programmes can facilitate the access to mental illness prevention. All stakeholder groups highlighted the possibility to use programmes independently of (i) ‘time’ and (ii) ‘location’. Thus, usage is perceived as more flexible and easier for people living in remote areas or hard to reach TGs without sufficient mental healthcare infrastructure or for people with tight schedules. Online programmes are considered to be offered at (iii) ‘low or no costs’ once they have been established. Furthermore, they can be easily translated and offered in (iv) ‘different languages’. This lowers the barriers on the practical side.

##### Low psychological barriers

On the psychological side, (i) ‘anonymity’ and (ii) the ‘absence of personal contact’ lowers the barriers for the use of programmes linked to mental health. In the context of a stigmatized topic like mental health, staying anonymous can be important. Since online interventions can be used ‘at home, where nobody is watching’ (TG) and users therefore cannot be ‘seen when visiting a psychiatrist or psychologist’ (TG), ‘stigmatization (…) is less of a topic with a tool that can be used anonymously’ (PM). Similarly, the absence of personal contact in online programmes can help to reach people who have problems talking to others, different psychological inhibitions (e.g. shame) or even suffer from social phobias. For those, it ‘might be an easier way than having a person with whom they have to learn to get along with’ (PM) and they can be ‘reached without fearing the reaction of the counterpart’ (F).

##### Psychoeducation

The stakeholders discussed that, if more people get easy access to information about mental health, on the long run this could (i) ‘reduce stigmatization’ and ‘contribute a lot to a new knowledge and a new awareness in society and support in general, for example that a neighbour doesn’t think ‘my neighbour is totally nuts’ anymore, because now he knows how someone acts who doesn’t feel good’ (TG).

The mere transfer of information does not require face-to-face counselling by fully trained professionals but can be also provided through high-quality online materials and tools. Getting in touch with this topic online could furthermore help people to figure out if they are potentially at risk of developing a mental disorder or even already have one. Hence, online prevention programmes could be an (ii) ‘entry to professional help’, e.g. when people receive feedback on assessment results. Strategies for self-help can be provided or affected people can receive addresses or links to professional treatment and can be encouraged to look for professional help: ‘Maybe then more people become aware of it (…) that they burden themselves with too much and have to set boundaries’ (TG).

##### Special features of digital technologies

When compared with conventional face-to-face mental illness prevention programmes, digital technologies are considered to offer new opportunities. Programmes can be (i) ‘individualized’ and adapted to the users’ needs or by the users’ choice ‘depending on their profile’ (PM). (ii) ‘Quantification tools’ like tracking and monitoring could also be used to gain objective information on the users’ health status, e.g. ‘if vital signs are monitored by wearables, in the future in the case of a stress reaction it might be possible to analyze what was the trigger?’ (TG). (iii) The content of the programme can be easily ‘updated’.

#### Disadvantages

##### Lack of personal contact

The lack of personal contact was not only perceived as advantage, but was also the by far most discussed disadvantage in the use of online programmes. Four main subthemes are perceived as a consequence of this:

Although we asked about prevention, the topic of developing a (therapeutic) relationship came up, associated with trust, human warmth, communication, and reciprocity. The (i) ‘lack of relationship’ in online programmes is therefore seen as disadvantage *per se*. (ii) ‘Lack of commitment’ is seen as another consequence, because ‘it is way easier to discontinue’ (PM). The downside of flexibility is the risk that users can ‘keep on delaying it’ (TG). By setting appointments with real persons, users ‘feel more obligation to go there and do something’ (TG). Therefore, the use of online programmes is considered to require more self-responsibility from the users. (iii) ‘Misunderstandings’ can be another consequence e.g. because ‘body language, which might say something else than the written word, could not be taken into account’ (F). The missing possibility to ask questions synchronously may lead to misunderstandings of the programme content because ‘there is no feedback in order to assure that you understood it, instead everyone understands what he wants to understand or what he is able to understand and that can be quite a gap’ (TG). The use of online programmes is (iv) ‘limited in more severe’ cases and might not be indicated for people with severe problems. Although online programmes could function as entry to professional treatment for—otherwise hard to reach—people with severe problems, online prevention could also hinder them to enter professional face-to-face treatment, making them think ‘ok, I can deal with it on the Internet, I don’t have to visit a psychologist or ask for personal advice’ (TG).

##### Internet-related issues

Online interventions are ‘only available for people with access to the Internet’. Even with access to the Internet, not everyone is able and willing to use digital technology for mental health purposes and this delivery method implies a ‘dependency on devices’ (F). Furthermore, it can be harmful to use ‘forums—for example for eating disorders—where users goad each other’ (TG). Moreover, the delivery via Internet implies trust concerns: ‘Data protection’ is especially important when sensitive information is provided by the user and ‘when there is nobody who declared obligation to maintain secrecy’ (TG). Also, the Internet is full of unserious offers, and the ‘integrity’ of a provider is often hard to evaluate by users, who bear a risk to ‘fall into wrong hands’ (TG).

Potential facilitators/delivery staff believed that the advantages of online prevention programmes would outweigh the disadvantages (mean = 1.53, SD = 2.37, median = 2) compared with face-to-face contacts, whereby facilitators/delivery staff in Switzerland and Spain see Internet-based mental illness prevention as more advantageous than facilitators/delivery staff in Austria and Germany (Kruskal–Wallis *H* = 18.96, *P* < 0.001). Furthermore, potential facilitators/delivery staff from different countries significantly differ in the extent they would be in favour of integrating online mental health programmes (Kruskal–Wallis *H* = 13.01, *P* = 0.005) and actively support the integration (Kruskal–Wallis *H* = 16.37, *P* = 0.001), with Switzerland and Spain expressing the most positive attitudes and Austria and Germany expressing less positive attitudes (Supplementary figure 1).

### Underserved groups

According to the participating stakeholders, many different groups might benefit from Internet-based prevention programmes for different reasons. People with psychological inhibitions (e.g. shame) might benefit from the anonymity, people with limited access to the healthcare system (e.g. living in rural areas or with limited mobility) might benefit from the independence of time and location. Young people might benefit because they are most familiar with digital technologies, and most preventive interventions should start early in life. Furthermore, people in critical life situations or exposed to risk factors, and relatives of mentally ill people are perceived as potential beneficiaries.

### Needs

#### Topics

Since we recruited a diverse range of stakeholders, a broad variety of topics that online prevention programmes should focus on, emerged. Four overarching themes were found, ranging from (i) ‘skills and healthy lifestyle’ (e.g. exercise, nutrition, relaxation and social skills) to (ii) ‘transitions and life events’ (e.g. career start, transition to retirement, mourning and migration), to (ii) ‘acute or chronic conditions associated with mental health problems’ (e.g. low self-esteem, stress, family problems or mobbing) and to (iv) ‘mental disorders’ (e.g. depression, anxiety, burnout, addiction, eating disorders, transdiagnostic disorders and psychoeducation about mental health). Facilitators’/delivery staff’s mean ratings regarding the relevance for pre-defined topics are depicted in Supplementary figure 2.

#### Aims

Overarching aims that should be achieved with online prevention programmes include (i) ‘mental health promotion and prevention’, (ii) ‘raising awareness’ for mental health issues by psychoeducation and de-stigmatization, (iii) ‘clarification about one’s own mental health status’ through self-assessments, and (iv) ‘referral to professionals if needed’, so that online prevention could function as first step into mental healthcare.

#### Desired characteristics of online mental illness prevention

We asked stakeholders which features an online mental illness prevention programme should contain and what it should look like. The answers can be divided into six main themes.

##### Development

Prior to the implementation of an online programme, three aspects should be considered in the development process: It should be (i) ‘evidence-based’ and based on existing established programmes (‘you imitate the good, remove the bad, improve it and adapt it for what we need’ (TG)), (ii) developed with the ‘participation’ of relevant TGs, and should appear (iii) ‘credible’, e.g. by showing a certification or by being supported by a reputable institution like a university and by not containing advertising.

##### General conditions

This theme refers to technical or other programme characteristics and includes (i) ‘usability’, (ii) ‘data protection and anonymity’, (iii) a ‘responsive webdesign’ for usage on different devices, (iv) the provision in ‘different languages’, (v) availability at ‘low or no costs’ and (vi) ‘recognizability of benefits and effectiveness’ by the users.

##### Presentation of content

These characteristics refer to the way the programme content is presented. Content, language and layout should be (i) ‘TGs adequate’, meaning interesting, attractive and appropriate to the respective TGs. Programme usage must be (ii) ‘customizable’ in terms of content, extent and transfer mode, since ‘some need (…) a diary, some just want to read information (…), some need case examples’ (TG). Furthermore, (iii) ‘positive framing’ means focussing on ‘wellbeing, not mental illness’ (TG), avoiding pathologizing language and rather conveying ‘humour and a certain straightforwardness (…) to make it fun to engage with the topic’ (PM).

##### Media and tools

A broad variety of tools for transferring the programme content was mentioned to keep users engaged. This includes (i) the use of ‘diverse media’ besides text, like pictures, videos or podcasts; (ii) ‘gamification tools’ like tracking, challenges, barometers, targets, ‘little tasks for the day (…) that you can tick off and thereby feel better’ (TG), ‘diaries targeting specific topics’ (TG) and (iii) ‘other features’ like self-assessments, links, addresses, newsletters, experience stories or follow-ups.

##### Contact options

The stakeholder groups discussed the benefits of enabling contact (i) with other users or ‘peers via’ forums, chats or other modes of exchange, because ‘sometimes it helps to realize that you are not alone with your problems and that you are able to discuss them with others’ (TG). Since negative comments in forums may emerge, forum moderation was suggested. Furthermore, (ii) ‘professionals working for the programme’ should be available via chats, messages or video calls, e.g. in acute situations or if there are further questions, so ‘that it is more like a conversation with a real person and not just with a computer analyzing data’. (TG). (iii) ‘Blended approaches’ were also mentioned as an option to combine the advantages of online and face-to-face sessions.

##### Motivators for participation

To improve adherence, e.g. (i) ‘external motivators’ [e.g. ‘incentive schemes’ or other benefits for health insurance (TG) or money], (ii) ‘internal motivators’ [e.g. motivational messages, reminders, gamification tools or perceiving the programme as having ‘a clear benefit for the users’ (TG)] and (iii) certain degrees of ‘obligation’ [e.g. a recommended minimum term or providing a ‘benchmark’ (TG)] were discussed.

The potential facilitators/delivery staff were additionally asked in the online questionnaire to rate the relevance of predefined characteristics for online prevention programmes in the field of mental health on a 10-point scale. The mean ratings are depicted in Supplementary figure 3. All characteristics were regarded as moderate to very important with data security, usability and ease of access showing the highest ratings and reminder functions, communication tools and personalized feedback showing the lowest ratings.

### Influencing factors alongside the RE-AIM dimensions

Stakeholders were asked about possible fostering and hindering factors for reach, adoption, implementation and maintenance of online mental illness prevention programmes in the healthcare setting.

#### Reach

The discussed topics concerning reach can be classified into two overarching themes:

1. ‘Potential users need to be aware that a programme exists’. This can be achieved through (i) ‘promotion’ via different channels, e.g. social media, the Internet in general, or via cross medial campaigns; but also through (ii) ‘support by health professionals’, e.g. recommendations of general practitioners to use the programme.
2. ‘The programme needs to be attractive’. Once users know about the programme, it should keep people interested, so they try it and keep on using it themselves. The following basic conditions and programme characteristics (see also section ‘Needs’) are crucial: Programmes have to be easy to use, attractive, TG adequate, trustworthy, cheap or for free and avoid pathologizing language; data security has to be guaranteed and the benefit of using it should be recognizable.

#### Adoption

The mentioned factors influencing organizational adoption can be divided into three subthemes.

1. ‘Given circumstances’ include (i) the ‘technical infrastructure’, and (ii) ‘existing structures’ like laws.
2. ‘Attitudes’ include (i) ‘scepticism’ concerning technology, mental illness prevention or ethical issues, (ii) ‘technical affinity’, and (iii) the ‘perception’ of online programmes ‘as competitors to replace face-to-face-interventions’.
3. ‘Modifiable factors’ refer to ‘programme characteristics’ and context factors. (i) Relevant programme characteristics include usability, data security, evidence-base and the overall programme quality. (ii) ‘Modifiable context factors’ can be influenced by researchers, providers or decision makers. It is crucial that potential facilitators/delivery staff and institutions receive enough information about the programme as well as training and support. The benefit both for the users and the institution should be recognizable. The integration of the programme into the daily routine should not only avoid additional workload but reduce existing workload instead, ideally without replacing health professionals but support them in their work. Finally, ‘economical aspects play an important role. You need resources, how it is funded, who pays for it? This is a crucial point, to provide solutions and possibilities, to employ resources’ (PM).

#### Implementation

Implementation refers to the delivery of the programme as intended and the required costs and time for the delivery. (i) ‘Training’ and (ii) ‘support’ for the potential facilitators/delivery staff are the key factors mentioned especially by PMs. Other factors additionally named by potential facilitators/delivery staff were (iii) ‘expenditure of time’, (iv) ‘usability’, (v) ‘coordination’ of implementation activities and (vi) ‘infrastructure’.

#### Maintenance

Three important factors which were assumed to influence maintenance were identified.

1. ‘Securing sustainable structures’. This includes secured financing, a person responsible for ongoing coordination of the programme implementation and embedding into the healthcare system.
2. Ongoing ‘efforts to ensure reach’; e.g. via promotion or support by decision makers like politicians.
3. Ongoing ‘evaluation and adaptation’ of programmes. Since the quality and effectiveness of online mental health programmes are crucial, they should be evaluated and improved continuously. Furthermore, benefits of the programme use must be apparent for the users.

## Discussion

In this study, we aimed to identify different stakeholder groups’ experiences, attitudes and needs towards online prevention in mental health in four European countries, perceived fostering and hindering factors for the reach, adoption, implementation and maintenance of such programmes and their integration into European healthcare systems as well as groups they consider as underserved and therefore most in need of online prevention.

The emerging themes across all stakeholder groups, countries and topics did not differ. This points to the positive fact, that there seems to be a common understanding regarding the implementation of online interventions to prevent mental health disorders into existing healthcare systems in Europe. Especially for a successful implementation of such programmes it can be extremely beneficial if PMs and potential facilitators/delivery staff understand and share the same needs and perceptions of these programmes as their TGs/potential users.

The results also indicate that attitudes towards their implementation were quite positive. However, among facilitators/delivery staff, attitudes were more positive in countries where also more experiences with online interventions were reported, like in Switzerland and Spain. Consequently, it can be assumed that raising awareness about these programmes can contribute to their acceptance. Likewise, awareness and acceptance have also proved to be relevant for reach, adoption and implementation of eMental Health initiatives.^8^^,^^10^^,^^28^

Stakeholders see potential in online programmes to increase mental health equality, since they can help to reach formerly underserved groups like people living in rural areas or with low income as already emphasized in another study.^29^ Specifically, stigmatization was discussed as big challenge for mental healthcare. The usage of face-to-face mental health interventions is often perceived as a sign of weakness. The absence of personal contact and the chance to stay anonymously are an advantage in this regard. Online prevention programmes could function as measures for psychoeducation in the general population and as first low-threshold entry point to professional help for individuals who are at risk of developing a mental health problem, or who have first symptoms.^30^

Despite the potential to increase reach due to anonymity, the absence of personal contact was also seen as a relevant disadvantage of online mental health programmes, especially, when it comes to commitment and the importance of relationships in cases with more pronounced symptoms which require intensive care. In previous studies the patient–professional relationship was also reported as very important aspect regarding online treatment for mental disorders.^10^^,^^16^ In this context, various contact options with professionals within the programme or the use of blended approaches combining Internet- and traditional face-to-face treatment components^31^ were described as desirable programme characteristics. This result is further supported by prior research stating that guided programmes increase adherence, motivation and commitment.^32^ However, research on whether uptake of and adherence to Internet-delivered psychological interventions can be increased by using blended approaches is scarce. Combining Internet-based approaches with face-to-face elements might as well result in lower uptake, as required face-to-face contacts might also be seen as a barrier for intervention participation. Additionally, various methods of interacting with other participants (e.g. online discussion groups) were discussed, which have shown to be related to increased engagement,^33^ the saving of resources^34^ and effectiveness.^35^ Moreover, as the results indicate, offering various interaction options (e.g. synchronic or asynchronic, generic or personal communication) might be an important asset in order to respond to the different needs and preferences of the TGs. For instance, in line with a stepped care approach the intensity of personal communication could be increased in relation to absence or presence of symptoms and user autonomy.^36^ However, in each step of such a stepped care approach generic communication (e.g. automated messages) must at least in case of (emergency) situations in which users need more intensive support be supplemented with personal communication from a real person.^37^

The results clearly show that quality assurance is a major issue, which is consistent with previous studies. The themes data protection, integrity and quality of online mental health programmes reflect these concerns. To tackle these issues, essential prerequisites, like a reliable data protection declaration, a certification that guarantees the quality and evidence-base of a programme, and a reputable provider have to be in place. Psychotherapists and clinical psychologists offering online interventions must adhere to laws regulating counselling and treatment in the field of mental health. Furthermore, over the past years there were several initiatives to develop quality standards for online interventions in Europe (e.g. FSP&FMPP, 2017^38^) which could serve as basis for certification. Other relevant characteristics like personalization,^32^ usability, adequateness for a given TG, the use of different media and feedback tools and evidence-base^35^ confirm findings of prior studies.

Our results indicate that multiple context factors have to be considered to successfully and sustainably integrate eMental Health technologies for prevention into existing healthcare systems. Some of these factors, like structures, funding and legal framework, cannot or hardly be influenced by researchers or suppliers, but by PMs. Therefore, their support and advocacy are essential. Additionally, the successful adoption and implementation of digital technologies is determined by the support and attitudes of potential multipliers (e.g. psychologists) who are needed to deliver or at least recommend the programmes to the TGs/potential users. To gain their support, these potential multipliers should receive information about eMental Health and training for their use. It has to be clearly communicated, that the integration of eMental Health could facilitate their work, without endangering their jobs. During implementation the offer of advice and support is important. These results are also in line with previous studies.^8^^,^^10^ The TGs/potential users should not be left out of consideration: Promotion is needed to make them aware of the existence and the potential benefits of such programmes,^30^ reduce common misperceptions and other barriers of intervention participation.^39^^,^^40^ Moreover, online mental health promotion and mental illness prevention programmes should be designed to meet the users’ needs and, very important according to the results of this study, the users should quickly recognize individual benefits in order to adhere to the programme.

Furthermore, the feel good and fun aspects mentioned by stakeholders are especially relevant in the prevention field, as the TGs usually have less psychological strain to use the programme compared with those of self-help and treatment. Attractiveness of the programme was considered as crucial by stakeholders. Similarly, Taylor *et al*.^30^ point out that reach, uptake and engagement are important variables for the optimization of outcome.

## Strengths and limitations

One of the key strengths of the ICare Stakeholder Survey is that it provides a multidimensional perspective regarding online interventions to prevent common mental health disorders by including different stakeholder groups and countries across Europe. However, since this Stakeholder Survey aimed to present results on two different levels, some methodical difficulties had to be taken into account. On the one hand, a comprehensive overview regarding online preventive mental health interventions across different stakeholder groups and countries was planned, and on the other hand, ICare partners had to be given the possibility to use the focus group results of the TGs/potential users for improving their individual interventions within their respective trials.^17^ This means, that we had to apply different inclusion criteria in different countries in accordance with the TGs/potential users of the various trials. However, since we considered all focus groups across countries and trials within our overall analysis and thus included a broad range of personal characteristics and situations, results can be aggregated and analyzed on a general level. Differences between countries in the quantitative responses given by potential facilitators/delivery staff in the online questionnaires must be interpreted with caution. Recruitment strategies were somewhat different in the countries, dependent on the individual situations. Convenience samples were used in the most countries, such that representativity of the samples might be affected in different ways. Additionally, it was not possible to calculate the response rate, since snowball principles were applied in recruiting. Participants across stakeholder groups had no detailed or shared understanding of the term ‘prevention’, although a definition was provided at the beginning of each questionnaire or conversation. Generally, participants associated rather pathological than salutogenic aspects with the term and sometimes mixed aspects of prevention and treatment. Furthermore, although the dimensions of the RE-AIM framework were introduced to the participants prior to each associated question, answers regarding the different dimensions overlapped. However, this may also imply that some of the mentioned facilitators or barriers are relevant to more than just one dimension.

## Conclusion

This study highlights aspects that need to be considered for the design and sustainable implementation of online mental illness prevention programmes in the healthcare setting. Apart from factors which are also relevant for treatment programmes (safety and data protection measures, trustworthy providers, evidence-base, appropriateness for TGs and delivery staff, awareness and acceptance in the TGs and delivery staff, policies, financial and staff resources…), key factors for preventive interventions are attractiveness, feel-good, fun and gamification elements in order to attract and engage the target audience. This seems especially relevant for users of preventive programmes, since it can be assumed that this TG is hard to reach and engage due to less psychological strain and the lack of motivation and perseverance to change something in their lives. The consideration of the herein before mentioned aspects is most certainly a challenge for existing healthcare systems. However, a lot of resources are already available and not providing preventive interventions can also be seen as a lost opportunity. Moreover, our results are also in line with recent research stating that e.g. screenings in preventive interventions can also be an important entry point for treatment.^30^

## Supplementary data

Supplementary data are available at *EURPUB* online.

## Funding

Funding for the study was provided by European Union’s Horizon 2020 research and innovation programme under grant agreement No 634757.

*Conflicts of interest*: None declared.

**Key points**

- In comparison to online treatment programmes, additional key factors for prevention programmes are attractiveness, feel-good, fun and gamification elements in order to attract this hard to reach target audience.
- The low structural and psychological barriers associated with online mental illness prevention can improve reach of former underserved populations and prepone the entry into professional mental healthcare.
- The lack of face-to-face contact and trust concerns are perceived disadvantages that can be combated by offering various interaction options depending on users’ severity of symptoms and autonomy and by putting transparent quality assurance measures into place.
- To guarantee a sustainable integration into healthcare systems, support and training for potential facilitators/delivery staff, ongoing efforts to ensure reach, and provision of detailed information on programme usage and benefits is key.

## Supplementary Material

ckab043_Supplementary_Material
