# Supplementary material for: Stakeholders’ views on online interventions to prevent common mental health disorders in adults implemented into existing healthcare systems in Europe
Source: Eur J Public Health. 2021 Jul 7;31(Suppl 1):i55–63. doi: 10.1093/eurpub/ckab043 (PMC8495679; doi:10.1093/eurpub/ckab043)
Supplement: ckab043_Supplementary_Material [file ckab043_supplementary_material.pdf]

Supplementary material

Kuso S, Nitsch M, Zeiler M, et al. Stakeholders' views on online interventions to prevent common mental health disorders in adults implemented into existing healthcare systems in Europe.

Table 2: Experiences of potential facilitators/ delivery staff with Internet-based prevention programmes by country (obtained in the online questionnaire)

Country → ↓ Type of experience	DE (%)	AT (%)	CH (%)	ES (%)	Chi^2, p-value	Significant post-hoc differences
Read or heard about such programmes	79.1%	69.0%	92.3%	91.3%	$\text{Chi}^2 = 8.97$, $p = .030$	AT < DE, CH, ES
Looked at such programmes	44.2%	26.8%	80.8%	60.9%	$\text{Chi}^2 = 25.36$, $p < .001$	AT < CH, ES<br>DE < CH
Used such programmes	7.0%	5.6%	30.8%	17.4%	$\text{Chi}^2 = 13.37$, $p = .004$	DE, AT, ES < CH
Implemented such programmes	20.9%	5.6%	30.8%	56.5%	$\text{Chi}^2 = 29.24$, $p < .001$	AT < DE, CH, ES<br>DE < ES
Counselled implementation of such programmes	14.0%	7.1%	42.3%	56.5%	$\text{Chi}^2 = 33.27$, $p < .001$	DE, AT < CH, ES
Online Interventions available in setting (% yes)	48.8%	11.3%	73.1%	8.7%	$\text{Chi}^2 = 63.65$ $p < .001$	AT,ES < DE < CH

DE = Germany, AT = Austria, CH = Switzerland, ES = Spain

FIGURES

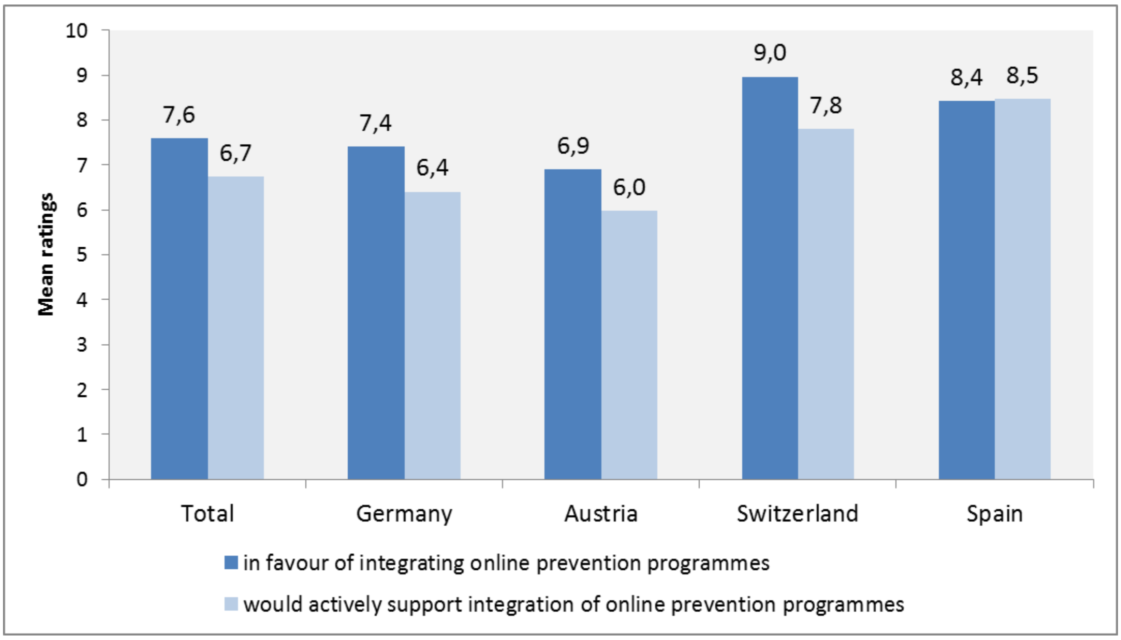

Figure 1: Mean ratings regarding the extent potential facilitators would be in favour of integrating and actively support the integration of Internet-based prevention programmes in the healthcare setting (0 = not at all, 10 = absolutely) (obtained in the online questionnaire)

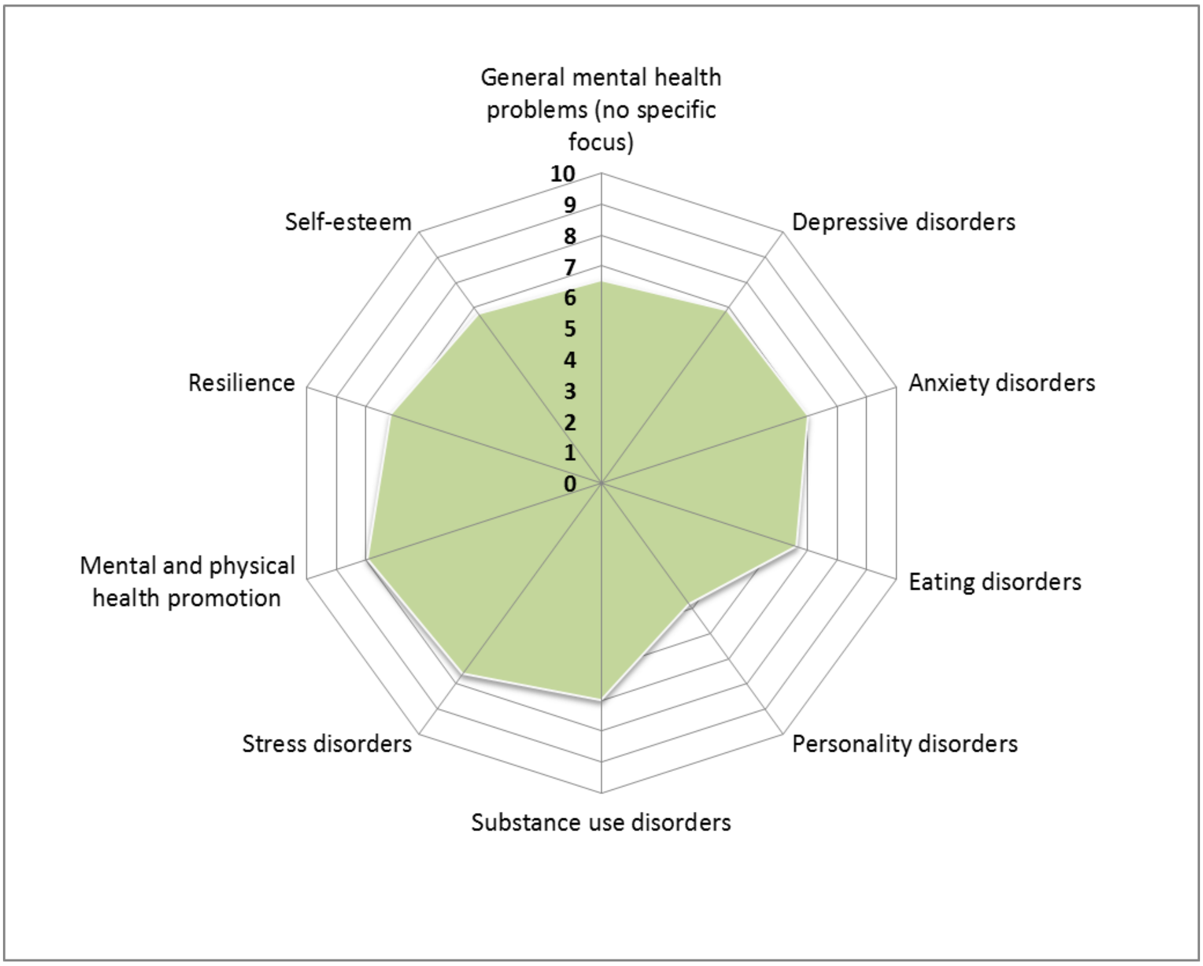

Figure 2: Mean ratings of potential facilitators/ delivery staff regarding the relevance of different topics for Internet-based prevention programmes in the healthcare setting (0 = not at all relevant, 10 = very relevant) (obtained in the online questionnaire)

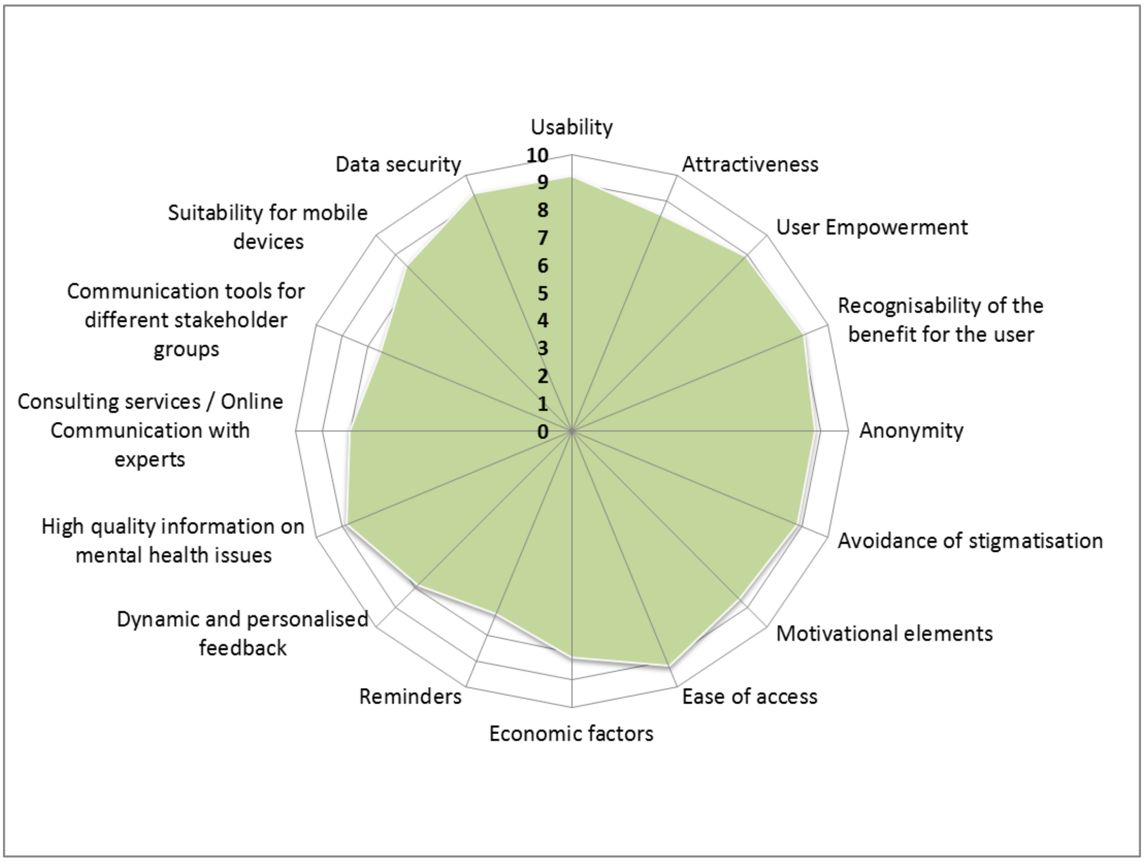

Figure 3: Mean ratings of potential facilitators/ delivery staff regarding the relevance of different characteristics of Internet-based prevention programmes in the healthcare setting (0 = not at all relevant, 10 = very relevant) (obtained in the online questionnaire)

I CARE

GA 634757

I CARE

Instruments

Stakeholder Survey Instruments

(Internet-based prevention in the field of mental health)

This project has received funding from the European Union's Horizon 2020 research and innovation programme under grant agreement No. 634757.

Table of Contents

1 Focus group topic guide (Target groups or Beneficiaries) ... 3
2 Semi-structured Interview Guide (Policy makers) ... 6
3 Online-Questionnaire (Facilitators or Enablers) ... 10

1 Focus group topic guide (Target groups or Beneficiaries)

Introduction

Welcome to our session. Thanks for taking the time to join us to talk about prevention in the field of mental health. My name is ….. and assisting me is …… We're researchers at the ….

At the moment we are working on a project, which is funded by the European Commission. The University of Dresden is leading the research project and researchers from 13 other research institutions and universities across Europe are participating. The aim of this project is to implement Internet-based interventions that aim to prevent mental health problems and disorders in different settings, such as universities, schools and the general health care system, across Europe. In order to successfully develop and implement these programs we would first like to explore the needs, values, attitudes and experiences of different stakeholder groups.

Thus, your views will provide valuable input for our programmes, so your opinion is very important to us. There are no wrong answers but rather differing points of view. Please feel free to share your point of view even if it differs from what others have said. Keep in mind that we are just as interested in negative comments as positive comments.

We are tape recording the session because we don't want to miss any of your comments. People often say very helpful things in these discussions and we can't write fast enough to get them all down. We won't use any names in our reports. You may be assured of complete confidentiality.

Before we can start we would need you to sign the consent form. Please read and sign it and let us know if you have any questions.

KEY TOPICS *[Add probes for fuller response and/or change order depending on situation and participants]*

Warm up (refers to "needs")

1. What do you think are the biggest mental health challenges for the health system in your country / for students within the first year at university / for pupils between 14 and 19?

[Relevance of topic "prevention in the field of mental health" in general]

2. How do you feel about prevention in the field of mental health in schools/universities/ the health care system in general?

[Experiences with Internet-based interventions]

3. What are your experiences with Internet-based interventions that aim to prevent mental health problems and disorders?
   a) Which Internet-based interventions that aim to prevent mental health problems and disorders are available for pupils at your school / students at your university/ in the health care system in your country? [Probe: name, age group, focus?]

[Values and attitudes]

4. What do you think about offering Internet-based interventions that aim to prevent mental health problems and disorders within the health care system in your country /in universities/in schools?
   a) What do you consider as the most important advantages of Internet-based interventions that aim to prevent mental health problems and disorders compared to face-to-face interventions?
   b) What do you consider as the most important disadvantages of Internet-based interventions that aim to prevent mental health problems and disorders compared to face-to-face interventions?

[Needs]

5. Which topics need to be focused on when offering Internet-based interventions that aim to prevent mental health problems and disorders in the health care system in your country /in universities/in schools?
   a) Which overall aims need to be focused on?
   b) Which characteristics would you consider important for Internet-based interventions that aim to prevent mental health problems and disorders in schools/universities/ the health care system in your country?
   c) Which groups of pupils / university students / individuals would you consider most underserved regarding Internet-based interventions that aim to prevent mental health problems and disorders?

*[Barriers and facilitators for reach, adoption, implementation, maintenance]**

[REACH]

6. Which factors need to be considered to make Internet-based interventions attractive (for pupils/ students)?

[IMPLEMENTATION]

7. Which factors need to be considered in order to successfully integrate Internet-based interventions that aim to prevent mental health problems and disorders in the daily (school/university) routine?

[CONCLUSION/ END]

8. Is there anything else you would like to tell us?

Thank the participants for their time. Reiterate that the discussions will remain confidential. Tell them that they are welcome to contact the research team to ask questions at a later date if they wish.

**level of adoption is only relevant on an organizational level, “maintenance” is covered by implementation; asking about “maintenance” and “future research” does not seem adequate at this early stage of the project.*

2 Semi-structured Interview Guide (Policy makers)

The following information will be pre-filled by the interviewer and will not be asked directly from the respondent:

1. Indicate Stakeholder Group (e.g. funders, government, health insurances, etc.)

2. Indicate level of influence (limited, medium, high)

3. Indicate country where currently based

4. Indicate type of interview (in-person vs. telephone)

Introduction

Thanks for taking the time to join us to talk about prevention in the field of mental health. My name is ….. (if applicable: and assisting me is ……) I am a researcher at the ….

At the moment we are working on a project, which is funded by the European Commission. The University of Dresden is leading the research project and researchers from 13 other research institutions and universities across Europe are participating. The aim of this project is to implement Internet-based interventions that prevent mental health problems and disorders in different settings, such as universities, schools and the health care system in general, across Europe. In order to successfully develop and implement these programs we would first like to explore the needs, values, attitudes and experiences of different stakeholder groups.

Your views will provide valuable input for our programmes, so your opinion is very important to us. Also, please keep in mind that we are just as interested in negative comments as positive comments.

We are tape recording the session because we don't want to miss any of your comments and we can't write fast enough to get them all down. We won't use any names in our reports. You may be assured of complete confidentiality.

Before we can start we would need you to sign the consent form. Please read and sign it and let us know if you have any questions.

KEY TOPICS *[Add probes for fuller response and/or change order depending on situation and interviewee]*

Warm up

1. Can you tell us a bit about your own work (if applicable: that focuses on mental health)?
   - What is the specific focus of your work (e.g. programme implementation or evaluation, policy)?
   - Can you give some examples of your work?
2. From your own experience in this sector, what would you say are the biggest mental health challenges for the health care system in your country / for students within the first year at university / for pupils between 14 and 19?

[Relevance of topic "prevention in the field of mental health" in general]

3. How do you feel about prevention in the field of mental health in schools/universities/ the health care system in general?
   - How relevant are interventions in this field?
   - Who should be responsible for initiatives regarding prevention in the field of mental health in the health care system / schools / universities?

[Underserved populations]

4. Which groups of pupils / university students / individuals would you consider most underserved regarding prevention of mental health problems and disorders?
   - How can those groups be reached?

[Experiences with Internet-based interventions]

5. What are your experiences with Internet-based interventions that aim to prevent mental health problems and disorders?
   - Which Internet-based interventions that prevent mental health problems and disorders are available for pupils at your school / university students at your university/ within the health care system in your country? [name, age group, focus]

[Needs]

6. Which topics need to be focused on when offering Internet-based interventions that aim to prevent mental health problems and disorders in schools/universities/ the health care system in your country?
   - Which overall aims need to be focused on?
   - Which characteristics would you consider important for Internet-based interventions that aim to prevent mental health problems and disorders in schools/universities/ the health care system in your country?

- Which groups of pupils / university students / individuals would you consider most underserved regarding Internet-based interventions that aim to prevent mental health problems and disorders?

[Values and attitudes]

7. What do you think about offering Internet-based interventions that aim to prevent mental health problems and disorders in schools/universities/ the health care system in your country?
   - What do you consider as the most important advantages of Internet-based interventions that aim to prevent mental health problems and disorders compared to face-to-face interventions?
   - What do you consider as the most important disadvantages of Internet-based interventions that aim to prevent mental health problems and disorders compared to face-to-face interventions?
8. Would you actively support the integration of Internet-based interventions that aim to prevent mental health problems and disorders in your school / your university / the health care system in your country?

[Barriers and facilitators for reach, adoption, implementation, and maintenance]

The following questions focus on potential **hindering and fostering context factors regarding different program phases**. In the research model that we are using, these phases are described as "reach, adoption, implementation and maintenance". In the following part we give you a short explanation about these phases and after that we ask you about potential barriers and facilitators that could apply to each of these phases.

[REACH]

<u>Definition:</u> **REACH** means the absolute number, proportion, and representativeness of individuals who are willing to participate in a given initiative.

9. Which factors could <u>foster</u> REACH of pupils / university students / individuals regarding the use of an Internet-based intervention program that aims to prevent mental health problems and disorders?
10. Which factors could <u>hinder</u> REACH of pupils / university students / individuals regarding the use of an Internet-based intervention program that aims to prevent mental health problems and disorders?

[ADOPTION]

<u>Definition:</u> **ADOPTION** means the absolute number, proportion, and representativeness of settings and intervention agents who are willing to initiate a program.

11. Which factors could <u>foster</u> the ADOPTION of Internet-based intervention programs that aim to prevent mental health problems and disorders in schools / universities / the health care system?

12. Which factors could hinder the ADOPTION of Internet-based intervention programs that aim to prevent mental health problems and disorders in schools / universities / the health care system?

[IMPLEMENTATION]

Definition: At the setting level, **IMPLEMENTATION** refers to the intervention agents' fidelity to the various elements of an intervention's protocol. This includes consistency of delivery as intended and the time and cost of the intervention.

13. Which factors could foster the IMPLEMENTATION of Internet-based intervention programs that aim to prevent mental health problems and disorders in schools / universities / the health care system?
14. Which factors could hinder the IMPLEMENTATION of Internet-based intervention programs that aim to prevent mental health problems and disorders in schools / universities / the health care system?
15. Who should be responsible for the IMPLEMENTATION of Internet-based interventions that aim to prevent mental health problems and disorders in schools / universities / the health care system?

[MAINTAINANCE]

Definition: **MAINTAINANCE** means the extent to which a program or policy becomes institutionalized or part of the routine organizational practices and policies. **MAINTAINANCE** is also known as sustainability.

16. Which fostering factors need to be considered that Internet-based interventions that aim to prevent mental health problems and disorders can be integrated in the regular practice of schools / universities / the health care system?
17. Which hindering factors need to be considered that Internet-based interventions that aim to prevent mental health problems and disorders can be integrated in the regular practice of schools / universities / the health care system?

[FUTURE RESEARCH]

18. What would you suggest for future research regarding interventions that prevent mental health problems and disorders in general?

[CONCLUSION/ END]

19. Is there anything else you would like to tell us?

Thank the participant for their time. Reiterate that the interview will remain confidential. Tell them that they are welcome to contact the research team to ask questions at a later date if they wish.

3 Online-Questionnaire (Facilitators or Enablers)

[INTRODUCTION]

Dear Participant,

ICare (Integrating Technology into Mental Health Care Delivery in Europe) is a project, which is funded by the European Commission. The University of Dresden is leading the research project and researchers from 13 other research institutions and universities across Europe are participating. The aim of this project is to implement Internet-based interventions that prevent mental health problems in different settings, such as universities, schools and the health care system in general, across Europe.

In order to successfully develop and implement these programs we would primarily like to explore the needs, values, attitudes and experiences of different stakeholder groups.

Your views will provide valuable input for our programs, so your opinion is very important to us.

This survey consists of 27 questions and will take about 30 minutes to complete. This survey is completely anonymous, we will not ask you to provide any information concerning your person and the organization you are working in.

Thank you for participating in this stakeholder survey!

Coordination:

TECHNISCHE UNIVERSITÄT DRESDEN

EPC European Project Center

Partners:

MEDICAL UNIVERSITY OF VIENNA

FERDINAND PORSCHE FERN FH

Universität Zürich UZH

ISGF

VU UNIVERSITY AMSTERDAM

KING'S College LONDON University of London

LSE

WESTFÄLISCHE WILHELMS-UNIVERSITÄT MÜNSTER

VNIVERSITAT DE VALÈNCIA

UNIVERSITAT JAUME·I

FAU FRIEDRICH-ALEXANDER UNIVERSITÄT ERLANGEN-NÜRNBERG

UNIVERSITÄT BERN

Advisory Committee:

STANFORD SCHOOL OF MEDICINE

Dachverband

THE UNIVERSITY OF MEMPHIS

This project has received funding from the European Union's Horizon 2020 research and innovation program under grant agreement No 634757.

[CHOOSE COUNTRY]

	**Please choose your country!**<br>**Bitte geben Sie Ihr Land an!**<br>**Por favor, elija su país!**<br>**Kies uw land!**
○	United Kingdom
○	Deutschland
○	Österreich
○	Schweiz
○	Nederland
○	Espana

[IF United Kingdom → English language version
IF Deutschland OR Österreich OR Schweiz → German language version
IF Nederland → Dutch language version
IF Espana → Spanish language version]

[FUNCTION]

**1.**	**Please indicate the setting of which you currently represent/ in which you are working at the moment** (If you represent/work (in) several settings, please choose the setting, (in) which you represent/work most of your time.)
○	**Health Care System** (health care professionals, e.g. general practitioners, psychiatrists, pediatricians, psychologists, psychotherapists, nurses)
○	**School System** (e.g. school teachers, school authorities, school psychological services, school counselling services, student representatives)
○	**University System** (e.g. university teachers , university authorities, university psychological services, university counselling services, university students association and groups, student representatives)

[IF Health Care System → Health Care System version
IF School → Version for School Setting
IF University → Version for University Setting]

1a.	**Please indicate your role** (If you have more than one role, please choose the role that you fulfil most of the time.)
○	Pupils' representative
○	School teacher
○	School principal
○	School psychologist
○	School physician
○	School social worker
○	Other function within the school setting
○	University student representative
○	University teacher / lecturer
○	Member of the university student association / group
○	University psychological / counselling service
○	Other function within the university setting
○	General practitioner
○	Pediatrician
○	Psychologist
○	Psychotherapist
○	Social Worker / Social Education Worker
○	Psychiatrist
○	Nurse
○	Other function within the health care system

1b.	**Please indicate the number of years of experience you have in the role chosen above.**

Numeric open answer

[IF "Other function within the school setting" in Question 1]:

**1c.**	**Which other role(s) within the school setting do you have?**
Open answer	

[IF "Other function within the university setting" in Question 1]:

**1d.**	**Which other role(s) within the university setting do you have?**
Open answer	

[IF "Other function within the health care system" in Question 1]:

**1e.**	**Which other role(s) within the health care system do you have?**
Open answer	

[A. Relevance of topic "prevention in the field of mental health" in general]

This questionnaire deals with prevention in the field of mental health. First of all, please read through what we understand by the term "prevention": By prevention, we mean all measures that are taken

- **Prior to the occurrence of mental health problems or**
- **When first symptoms of mental health problems occur**

In other words:
We are dealing with measures that help…

- **To prevent mental health problems or**
- **Prevent the progression of first symptoms.**

In the following, please think about such preventive measures for pupils/university students/ individuals in your country.

The next questions deal with your views on prevention in the field of mental health in general. By this, we mean all measures that are taken prior to the occurrence of mental health problems or when first symptoms occur.

2.	**To what extent do you personally consider prevention in the field of mental health relevant in for pupils in schools/ for students in universities/ the health care system in your country?**
Slider	not at all relevant — 0 1 2 3 4 5 6 7 8 9 10 — very relevant

3.	**To what extent do you consider your school / your university / the health care system responsible for prevention in the field of mental health for pupils / university students?**
Slider	not at all responsible — 0 1 2 3 4 5 6 7 8 9 10 — very responsible

4.	**To what extent do you in your role feel personally responsible for prevention initiatives for mental health for pupils / for students / in the health care system in your country?**
Slider	not at all responsible — 0 1 2 3 4 5 6 7 8 9 10 — very responsible

[B. Underserved populations]

5.	**On your opinion, which group(s) of pupils / university students / individuals might most benefit from prevention initiatives in the field of mental health?**
Group 1	*Open answer*
Group 2	*Open answer*
Group 3	*Open answer*

5a.	**How can those group(s) that you mentioned be best reached by prevention measures in the field of mental health in schools / universities / health care system?** *(Please give a short suggestion for each of the groups)*
[Fill in answer of Group 1 of Q5 here]	*Open answer*

[Fill in answer of Group 2 of Q5 here]	*Open answer*
[Fill in answer of Group 3 of Q5 here]	*Open answer*

The following part deals with Internet-based prevention programs in the field of mental health.

In contrast to conventional approaches, participants receive interactive and tailored information and exercises related to mental health via computer or mobile devices (smartphone, tablet). The interaction with coaches, moderators or experts as well as with other participants usually doesn't take place in person but via emails, online messages or discussion boards.

Used continuously over a certain period of time, Internet-based programs can contribute preventing mental health problems or progression of first symptoms.

Internet-based prevention programs are also called computer-based, web-based or online programs.

The following questions deal with your experiences, needs and attitudes regarding **Internet-based prevention programs** in the field of mental health.

[C. Experiences with Internet-based interventions]

**6.**	**Please state, if the following statements regarding Internet-based prevention programs in the field of mental health apply to you:**
○ ○<br>Yes No	I have read or heard about it.
○ ○<br>Yes No	I have had a look at such programs.
○ ○<br>Yes No	I have used such programs myself (to prevent own mental health problems).
○ ○<br>Yes No	I have implemented or helped to implement such programs (at a school, university, in the health care system or in a different setting).
○ ○<br>Yes No	I have counselled individuals using such programs as a coach, moderator, or an expert.

7.	**Are there any Internet-based prevention programs in the field of mental health available for pupils at your school / students at your university / individuals in the health care system in your country?**
○	*Yes*
○	*No*
○	*I'm not sure / I don't know*

[IF "YES "on question 7→ proceed with question 7a
IF "NO" or "I'm not sure" on question 7→ proceed with question 8]

7a. Please provide more details about these programs in the table below:

**Name of the program** (if available)	**Age group** Please provide the age group the program is targeted to; write "general" if the program is not targeted at a specific age	**Focus** Please state the focus of the program – e.g. depression, anxiety, eating problems; if there is no specific focus: write "general"	**Aim(s) of the program** please describe in short the aim(s) of the program, e.g. "enhancing self-esteem"
Open answer	*Open answer*	*Open answer*	*Open answer*
Open answer	*Open answer*	*Open answer*	*Open answer*
Open answer	*Open answer*	*Open answer*	*Open answer*
Open answer	*Open answer*	*Open answer*	*Open answer*
Open answer	*Open answer*	*Open answer*	*Open answer*

8.	**Altogether, <u>how much experience</u> do you have with Internet-based prevention programs in the field of mental health?**

Slider	No experience whatsoever 0 1 2 3 4 5 6 7 8 9 10 Very much experience

[D. Needs]

The next set of questions deal with topics, aspects and characteristics for Internet-based prevention programs in the field of mental health addressed to pupils at schools / students at universities / in the health care system in your country.

**9.**	**How relevant do you consider the following topics for Internet-based prevention programs in the field of mental health?**
General mental health problems (no specific focus)	not at all relevant 0 1 2 3 4 5 6 7 8 9 10 very relevant
Anxiety Disorders	not at all relevant 0 1 2 3 4 5 6 7 8 9 10 very relevant
Depressive Disorders	not at all relevant 0 1 2 3 4 5 6 7 8 9 10 very relevant
Eating Disorders	not at all relevant 0 1 2 3 4 5 6 7 8 9 10 very relevant
Personality Disorders (perception and behavior are not flexible and appropriate; e.g. excessive distrust, violation of social norms, persistent worries, etc.)	not at all relevant 0 1 2 3 4 5 6 7 8 9 10 very relevant
Substance Use Disorders (e.g. abuse of alcohol, drugs, nicotine)	not at all relevant 0 1 2 3 4 5 6 7 8 9 10 very relevant
Stress Disorders	not at all relevant 0 1 2 3 4 5 6 7 8 9 10 very relevant
Mental and physical health Promotion	not at all relevant 0 1 2 3 4 5 6 7 8 9 10 very relevant

Resilience(ability to cope with crises)	not at all relevant 0 1 2 3 4 5 6 7 8 9 10 very relevant
Self-esteem	not at all relevant 0 1 2 3 4 5 6 7 8 9 10 very relevant

**9a.**	**Which other topics would you consider very relevant?**
Open answer	

**10.**	**How relevant do you consider the following aspects for Internet-based prevention programs in the field of mental health addressed to pupils in schools / students in universities / in the health care system in your country?**
Prevent onset of mental health problems and disorders	not at all relevant 0 1 2 3 4 5 6 7 8 9 10 very relevant
Reduce risk factors for mental problems and health disorders	not at all relevant 0 1 2 3 4 5 6 7 8 9 10 very relevant
Increase protective factors	not at all relevant 0 1 2 3 4 5 6 7 8 9 10 very relevant
Prevent progression of first symptoms of mental disorders	not at all relevant 0 1 2 3 4 5 6 7 8 9 10 very relevant
Reduce severity of first symptoms of mental disorders	not at all relevant 0 1 2 3 4 5 6 7 8 9 10 very relevant

**11.**	**How relevant do you consider the following characteristics for such prevention programmes?**
Usability (e.g. easy to use)	not at all relevant 0 1 2 3 4 5 6 7 8 9 10 very relevant
Attractiveness (e.g. modern, appealing interface)	not at all relevant 0 1 2 3 4 5 6 7 8 9 10 very relevant

User Empowerment (competence for self-help)	not at all relevant 0 1 2 3 4 5 6 7 8 9 10 very relevant
Recognizability of the benefit for the user	not at all relevant 0 1 2 3 4 5 6 7 8 9 10 very relevant
Anonymity	not at all relevant 0 1 2 3 4 5 6 7 8 9 10 very relevant
Avoidance of stigmatization (reducing feelings of being prejudged and marginalized)	not at all relevant 0 1 2 3 4 5 6 7 8 9 10 very relevant
Motivational Elements (e.g. feedback, rewards)	not at all relevant 0 1 2 3 4 5 6 7 8 9 10 very relevant
Ease of access	not at all relevant 0 1 2 3 4 5 6 7 8 9 10 very relevant
Economic Factors (costs, time exposure)	not at all relevant 0 1 2 3 4 5 6 7 8 9 10 very relevant
Reminders(e.g. via SMS or email) to participate regularly until the end	not at all relevant 0 1 2 3 4 5 6 7 8 9 10 very relevant
Dynamic and Personalized Feedback (e.g. suggestions related to entered data)	not at all relevant 0 1 2 3 4 5 6 7 8 9 10 very relevant
High-quality Information on Mental Health Issues (comprehensible, professional)	not at all relevant 0 1 2 3 4 5 6 7 8 9 10 very relevant
Consulting Services / Online Communication with Experts (e.g. help line, e-mail support, discussion forum)	not at all relevant 0 1 2 3 4 5 6 7 8 9 10 very relevant
Communication Tools (e.g. closed discussion forum) for different stakeholder groups (e.g. teachers, pupils, students, clients, patients)	not at all relevant 0 1 2 3 4 5 6 7 8 9 10 very relevant

Suitability for Mobile Devices (smartphones, tablets)	not at all relevant 0 1 2 3 4 5 6 7 8 9 10 very relevant
Data Security	not at all relevant 0 1 2 3 4 5 6 7 8 9 10 very relevant

**11a.**	**Which <u>other characteristics</u> would you consider as very relevant?**
Open answer	

[E. Values and Attitudes]

**In your opinion, what are the three most important <u>advantages</u> and the three most important disadvantages of Internet-based prevention programs in the field of mental health, implemented in schools / universities / the health care system compared to face-to-face interventions?**

12. The three most important advantages:

1. Open answer
2. Open answer
3. Open answer

13. The three most important disadvantages:

1. Open answer
2. Open answer
3. Open answer

**14.**	**If you consider the advantages and disadvantages you have mentioned, how do you balance them in total?**
Slider	Much more disadvantages -5 -4 -3 -2 -1 0 1 2 3 4 5 Much more advantages

15.	**Would you be <u>in favor</u> of integrating Internet-based prevention programs in the field of mental health in your school / your university / the health care system in your country?**
Slider	not at all 0 1 2 3 4 5 6 7 8 9 10 absolutely

16.	**Would you <u>actively support</u> the integration of such programs in your school / your university / the health care system in your country?**
Slider	not at all 0 1 2 3 4 5 6 7 8 9 10 absolutely

[F. Barriers and facilitators for reach, adoption, implementation, and maintenance]

The following questions focus on potential **hindering and fostering context factors** for different program phases (**1. Willingness for Implementation (Adoption), 2. Reach, 3. Implementation, 4. Maintenance)**. You are asked to provide your own ideas, suggestions and recommendations. Although some questions might be difficult for you to answer, please try to give a short answer to each of them, as your suggestions are very important for the successful implementation of the ICare project and other upcoming projects.

1. Willingness for implementation (ADOPTION)

By **Willingness for Implementation (Adoption)** we mean the proportion of schools and school personnel (e.g. teachers, school authorities, school psychologists, school physicians, school social workers, etc.) / universities and university personnel (e.g. university teachers, university authorities, university psychological services, university counselling services, university students association and groups, student representatives) / facilities and professionals in the health care system (e.g. general practitioners, psychiatrists, pediatricians, psychologists, psychotherapists, nurses) who are willing to implement a program.

What factors could influence the willingness for implementation (ADOPTION) of Internet-based prevention programs in the field of mental health in schools / universities / the health care system in your country?

17.	**Fostering factors for the willingness of implementation (adoption) in schools / universities / the health care system in your country:**
Open answer	

**18.**	**Hindering factors for the willingness of implementation (adoption) in schools / universities / the health care system in your country:**
Open answer	

2. REACH

By **Reach** we mean the proportion of pupils /university students / individuals of the target group(s) who are willing to participate in a programme.

What factors could influence the reach of Internet-based prevention programs in the field of mental health?

**19.**	**Fostering factors for the reach in schools / universities / the health care system:**
Open answer	

**20.**	**Hindering factors for the reach in schools / universities / the health care system:**
Open answer	

3. IMPLEMENTATION

By **Implementation** we mean that a programme is implemented as intended by different personnel in schools (e.g. teachers, school authorities, school psychologists, school physicians, school social worker, etc.) / universities (e.g. university teachers, university authorities, university psychological services, university counselling services, university students association and groups, student representatives, etc.) / the health care system (e.g. general practitioners, psychiatrists, pediatricians, psychologists, psychotherapists, nurses).

What factors could influence the implementation of Internet-based prevention programs in the field of mental health in schools / universities / the health care system in your country?

**21.**	**Fostering factors for the implementation in schools / universities / the health care system:**
Open answer	

22. Hindering factors for the implementation in schools / universities / the health care system:
Open answer

23. Who should be responsible for the IMPLEMENTATION of such programs in schools / universities / the health care system in your country?
Open answer

4. MAINTAINANCE

By **Maintenance** we mean the sustainability of the programme in schools / universities / the health care system (i.e. that the programme can be offered and used continuously).

What factors could influence the maintenance of Internet-based prevention programs in the field of mental health in schools / universities / the health care system in your country?

24. Fostering factors for the maintenance in schools / universities / the health care system:
Open answer

25. Hindering factors for the maintenance in schools / universities / the health care system:
Open answer

[G. Future research]

26. What would you suggest for further research regarding prevention programs in the field of mental health in general?
Open answer

Further Comments:

**27.** **Is there anything else you would like to let us know?**
Open answer

Thank you very much for your participation in this survey! Your responses have been saved. Please close the browser window.
